# Coronary artery segmentation in angiographic videos utilizing spatial-temporal information

**DOI:** 10.1186/s12880-020-00509-9

**Published:** 2020-09-24

**Authors:** Lu Wang, Dongxue Liang, Xiaolei Yin, Jing Qiu, Zhiyun Yang, Junhui Xing, Jianzeng Dong, Zhaoyuan Ma

**Affiliations:** 1 The Future Laboratory, Tsinghua University, Beijing, 100084 China; 2 Department of Information Art and Design, Academy of Arts and Design, Tsinghua University, Beijing, 100084 China; 3 Center for Cardiology, Beijing Anzhen Hospital, Capital Medical University, Beijing, 100029 China; 4 The First Affiliated Hospital of Zhengzhou University, Zhengzhou, 450052 China

**Keywords:** Coronary artery angiography, Image segmentation, Video segmentation

## Abstract

**Background:**

Coronary artery angiography is an indispensable assistive technique for cardiac interventional surgery. Segmentation and extraction of blood vessels from coronary angiographic images or videos are very essential prerequisites for physicians to locate, assess and diagnose the plaques and stenosis in blood vessels.

**Methods:**

This article proposes a novel coronary artery segmentation framework that combines a three–dimensional (3D) convolutional input layer and a two–dimensional (2D) convolutional network. Instead of a single input image in the previous medical image segmentation applications, our framework accepts a sequence of coronary angiographic images as input, and outputs the clearest mask of segmentation result. The 3D input layer leverages the temporal information in the image sequence, and fuses the multiple images into more comprehensive 2D feature maps. The 2D convolutional network implements down–sampling encoders, up–sampling decoders, bottle–neck modules, and skip connections to accomplish the segmentation task.

**Results:**

The spatial–temporal model of this article obtains good segmentation results despite the poor quality of coronary angiographic video sequences, and outperforms the state–of–the–art techniques.

**Conclusions:**

The results justify that making full use of the spatial and temporal information in the image sequences will promote the analysis and understanding of the images in videos.

## Background

Physicians have been practicing interventional surgeries to diagnose and treat cardiovascular diseases for several decades. They locate, assess and diagnose the blood vessel stenosis and plaques by directly watching the angiographic videos with naked eyes during the surgeries. Based on their experiences, the physicians quickly make a qualitative judgment on the patient’s coronary artery condition and plan the treatment. This direct method is greatly affected by human factors and lacks accuracy, objectivity and consistency. Automated cardiovascular segmentation will help reduce the diagnostic inaccuracies for physicians. Many blood vessel extraction methods based on image segmentation have emerged driven by this motivation. Recently, with the development of deep learning, various deep neural network architectures have been proposed and applied in the medical image segmentation field [1–4]. Early deep learning–based approaches used the image patches and a sliding window block to traverse the image [5]. But the sliding window method casts a huge amount of computation, and misses the global contexts of the image at the same time. Yang et al. [6] used two convolutional neural networks (CNN) to process the patches and the whole image to obtained good performance in the segmentation of coronary artery vessels in angiograms, but it is very time–consuming. In 2015, fully–convolutional network [7], encoder–decoder network [8], and U–Net [9] were proposed and achieved good results. Since then, various methods based on U–Net architecture have sprung up. M–Net added a multi–scale input image and deep supervision [10]. New modules have been proposed to replace some blocks in the U–Net architecture to enhance the feature learning ability. Gibson et al., proposed a dense connection in the encoder block [11]. Zhao et al., introduced a modified U–Net by adding a spatial pyramid pooling [12]. Gu et al., inserted a dense atrous convolution (DAC) block and a residual multi–kernel pooling (RMP) block into the bottle–neck part, to extract and preserve more spatial context, and this it the state–of–the–art model, the CE–Net [13]. Zhang et al. introduced an attention-guided network to improve the performance of retina vessel segmentation [14]. A dual encoding U–Net was proposed to replace the skip–connections with attention modules to further promote of retinal vessel segmentation [15].

Nevertheless, some essential problems are associated with the blood vessel segmentation tasks in cardiovascular angiographic images. First, the shape of the cardiac blood vessels is complex and easily deformed. Coronary arteries have a tubular curved structure, and some can block, cover or entangle with one another, making the semantic information confusing in the images. Second, the angiographic images contain not only blood vessels, but also other organs and tissues which have similar shape and grayscale values, making it even more difficult to correctly extract the object [16]. Third, in order to minimize the damage to the health of the patients and physicians during the surgery, it is inevitable to reduce the dose of X–rays [17, 18], which results in low illumination and insufficient signal–to-noise ratio of the images [19], making the segmentation tasks even more challenging. Considering that the angiographic video is composed of a series of time–continuous image sequences, combining and processing several consecutive frames of images may provide a good insight for solving these problems. Intuitively, the blood vessels inter-blocking each other in one frame may be separated in another one. The problem of low signal–to–noise ratio caused by low illumination may be eliminated by the accumulation of multiple images. Therefore, the temporal dimension of the video also contains rich contextual information. Utilizing the time domain information to segment blood vessels from angiographic videos becomes a topic worthy of study.

In the mean time, since 2D CNN has achieved good results in image processing tasks, researchers have extended their interests to the video classification and segmentation field. The temporal information in the video is regarded as the third dimension, and 3D CNN is introduced as an important tool. The Inception 3D (I3D) architecture proposed by Carreira et al. is one of the pioneer 3D models [20], which inflates all the 2D convolutional kernels in the Inception V1 architecture [21] into 3D kernels, and is trained on the large–scale Kinetics dataset of videos [22]. However, the computational cost of 3D convolution is extremely high, so a variety of mixed convolutional models based on ResNet architecture have been proposed to resolve this dilemma. Another way to reduce the computational cost is to replace the 3D convolution with separable convolutions. In order to effectively make use of the temporal dimension, [23] proposed a R(2+1)D convolutional neural network, and [24] proposed a model called the pseudo 3D network.

In this article, we consider combining the advantages of 3D and 2D convolution to accomplish the task of blood vessel segmentation from coronary angiographic videos. Based on the architecture of 2D U–Net and its derivative CE–Net, we propose a 3D–2D network. The 3D convolutional layer mainly serves to extract and process temporal information in the video, and the 2D network extracts the spatial information. Our main contributions are as follows:

- A novel deep learning architecture combining 3D and 2D convolution to extract both temporal and spatial information from videos.
- A new application of deep learning–based video segmentation algorithm in the medical imaging field.

## Methods

In this section, the role of 3D convolution in volumetric image segmentation, and in video processing is investigated briefly. Then we give a detailed elaboration of the 3D layer and the 2D network design, and the determination of an important hyper–parameter in the end.

### Volumetric images and image sequence

3D convolution has been widely used in the volumetric medical image processing, such as tumor segmentation from layered scanned organic computerized tomography (CT) [25, 26]. The CT renders a series of layered 2D images, and the layers are stacked into a 3D volumetric image. The physical meaning of the spatial dimensions (width, height, depth) of this volumetric image is very clear, and the correlation between the layers constitutes the spatial context information of the image data. Therefore, it is a natural approach to apply 3D convolutional networks to volumetric image segmentation tasks.

However, coronary angiography is fundamentally different from CT scan imaging. Coronary angiography obtains a two–dimensional image whose spatial depth information has been squeezed. There are only two spatial dimensions, ie., width and height. The traditional coronary artery segmentation task is to process an independent contrast image, and a 2D convolutional neural network is used to extract the spatial details and context information from the image. We cannot directly apply 3D convolutional networks to such image segmentation tasks. It should be noted that coronary angiography is a continuous imaging process, and the sequence of moving contrast images forms a video. Thus the video contains information in three dimensions, ie., two spatial dimensions, and one temporal dimension. Previous work proved that the temporal dimension in the videos contains rich image semantic representations [23]. Making full use of the temporal information can greatly benefit image semantic segmentation, and enhance the performance of tasks such as scene understanding and target recognition. 3D convolutional networks have been widely applied in these tasks.

### 3D–2D network

This article is inspired by the work of deep learning based video processing and 2D medical image segmentation methods. The sequential frames of the coronary angiographic video are stacked to form 3D data. We use a combination of 3D convolutional layers and a 2D network for the blood vessel segmentation task. The 3D and 2D convolutions are not simply combined applications, but each has its own purpose, and the order of the two operations cannot be reversed.

#### 3D input layer

**Fig. 1:**
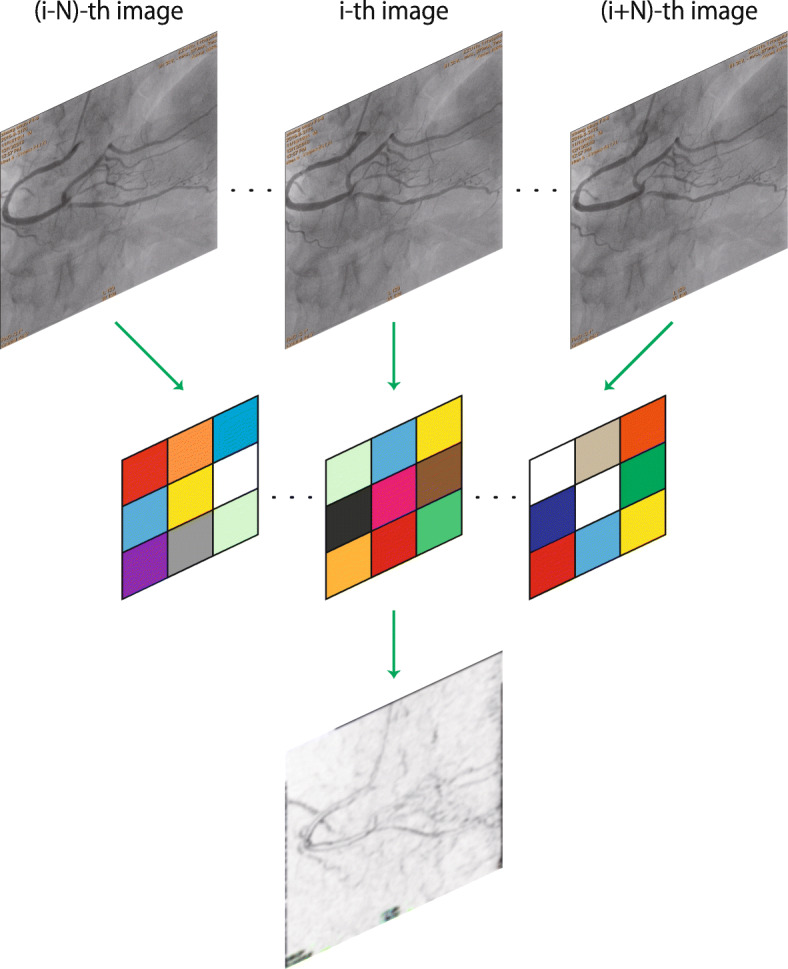
3D convolution for image sequences. With zero paddings in the *z*−axis, the 3D convolution transforms the 3D input image data into several channels of 2D feature maps

In our design, the network accepts several consecutive frames of image as input, and then outputs the segmentation mask corresponding to the middle frame. This is a task where multiple inputs correspond to one output, and the transformation of the number of images is implemented by the 3D input layer. We define the image to be segmented as the target image, and the temporally adjacent 2*N* images are referred to as auxiliary images, which contain *N* images before and after the target image, respectively. The 2*N*+1 input images form 3D data with a resolution of (2*N*+1)×*W*×*H*. The spatial dimensions of the 3D data is annotated as *x* and *y*, and the temporal dimension as *z*. The 3D input data are extracted and fused by the 3D convolution layer to obtain several channels of 2D feature maps, with a resolution of *W*×*H*, whereas the depth of *z* reduced to 1. Dimension reduction is achieved by a special design of the 3D convolutional kernel, that is, the size of the 3D kernel is (2*N*+1)×3×3, the paddings in the *x*−,*y*− and *z*− axes are 1, 1, 0, respectively, and the strides are all 1 in the three axes. A brief illustration of the 3D convolution operated on image sequences is given in Fig. 1 and Eq. 1.

1 $$ g(x,y) = \sum_{t=-N}^{N} \sum_{w=-1}^{1} \sum_{h=-1}^{1} w(t, w, h)f(i+t, x+w, y+h),   $$

where the i-th image in the sequence is the target image, and the neighboring 2*N* images are taken into account for the convolution in the *z*−axis.

Because the 3D convolutional layer has a small kernel size in the spatial dimensions *x* and *y*, the receptive field in the spatial dimensions is limited. As a consequence, the 3D convolutional operation is mainly used to extract and fuse the time domain information and the temporal correlations between successive frames in the video. Weighted combinations of the pixel values of the multi–frame images are then merged into 2D feature maps. Another 2D convolutional layer followed by a 2D max pooling layer is inserted right after the input 3D layer, to adjust the channel number as well as reducing the resolution of the feature maps to 1/2, to fit the dimensions of the first encoder module of the subsequent 2D network.

#### 2D network

The 2D CE–Net [13], which is composed of down–sampling encoder modules, up–sampling decoder modules, bottle–neck (DAC and RMP modules), and skip–connections, is used as the backbone network in our architecture. It is intended to extract several levels of spatial correlation in different scales in the fused 2D feature maps, and identify the foreground and the background pixels in the target image.

#### Network architecture

**Fig. 2:**
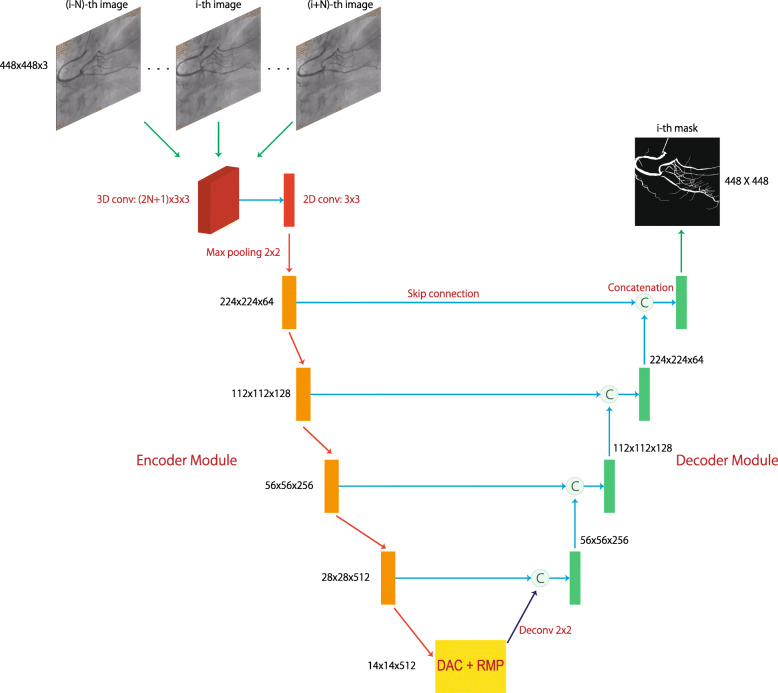
Network architecture. The input layer of the network is a 3D convolutional layer which has a kernel size of (2*N*+1)×3×3, and it accepts 2*N*+1 adjacent frames and outputs 2D feature maps of 16 channels, which are further fused by a 2D convolutional layer. The subsequent part of the network is analogous to 2D CE–Net. The network outputs a single mask image corresponding to the central frame of the input. In practice, *N* is usually set to 1 or 2

**Fig. 3:**
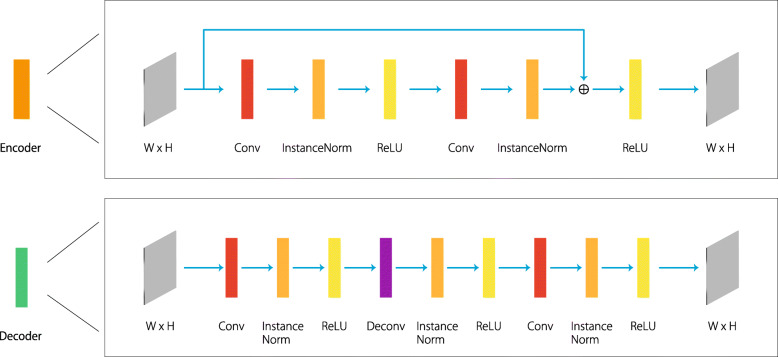
Structures of the encoder and decoder modules

The network architecture, as well as the input image sequence and the output mask are illustrated in Fig. 2. The detailed structures of the encoder and decoder are illustrated in Fig. 3. The main innovation and contribution of this article lie in the use of 3D and 2D convolutions to process different domains of information, as explained earlier, as well as the limitation of the computational complexity to an acceptable range by this 3D and 2D hybrid network design, which is going to be elaborated in the “Results” section.

#### Choice of *N*

As the heart beats cyclically, the cardiovascular vessels expand and contract in cycles accordingly. In the process of vessel motion, mutual blocking between blood vessels and deformations will inevitably occur. The value of *N* is an important hyperparameter. The smaller *N* is, the fewer input neighboring images are captured, and the extracted temporal context information is confined to a small range, but more accurate spatial details are retained. The larger the value of *N* is, the more adjacent images are input into the network, and the broader the temporal context information extraction and fusion is. The larger value of *N* keeps a long–term trend of motion and has a positive significance for eliminating the occlusion of vessels due to vascular motion. Considering that the imaging frame rate of existing coronary angiography equipments is relatively low, generally ranging from 10 to 15 frames per second (*fps*), and the cardiac cycle is generally smaller than one second, a large value of *N* will introduce a lot of problems. For instance, if we set *N* to 3, a total number of seven adjacent images will be input into the network, which cover more than a half cardiac cycle. This will undermine the significance of the target image in the image sequence, and increase the computational complexity. So we set the value of *N* to 1 or 2, that is, the network accepts 3 or 5 adjacent frames as input.

## Results

### Dataset

In collaboration with hospitals, we collected 170 coronary artery angiography video clips of cardiac interventional surgeries. The length of each video clip ranges from 3 to 10 seconds, recording the process from the injection of the contrast agent to the flow, diffusion and gradual disappearance of the contrast agent in the blood vessels. These videos are desensitized to hide the patient’s personal information and protect the patient’s right to privacy. The acquisition equipments of these video clips are products of many different manufacturers, so the image resolution ranges from 512×512 to 769×718, the frame rate ranges from 10*fps* to 15*fps*, and the original video format is wmv, MP4, etc. We use ffmpeg software toolkit to extract each frame of the videos and save it as a losslessly encoded red–green–blue (RGB) image file with a color depth of 8 bits in each chromatic channel. The total number of the source images is 8,835. We invite students from several medical colleges to manually segment and label the coronary arteries in these source images to generate a label image with an identical resolution of each image. Thus we have obtained a dataset containing 8,835 source images and 8,835 label images, which is named the CadVidSet.

### Partition of the training and test sets

The CadVidSet dataset is comprised of 170 sub–paths, each containing an average of 52 source images and 52 label images, and the images are stored in the chronological order of each video clip. We divide the images of each sub-path into a training set and a test set, where the training set accounts for 5/6 of the total images and the test set accounts for the other 1/6. As the input layer of the network accepts several time–continuous images, the division method is no longer a random selection of a single image, but the first 1/6 of each subpath is used as the test set, and the last 5/6 is used as the training set, or in the reverse order. The two division methods are randomly determined when the training process begins. This hard–cutting partitioning method avoids the error that an image appears in both the training and test sets. In order to ensure that each image is used in training or test, we have padded the training and test sets according to the value of *N* in the network input layer. The first image and the last image in each subset are copied *N* times to achieve a similar effect as the padding in the convolutional operations.

### Performance metrics

In training the model, we use the combined loss of dice loss and regularization loss as the objective function. We also calculate the IOU (intersection over union) to evaluate the performance in both training and test stages. Dice coefficient and IOU are both metrics on the similarity between two sets, with slight differences,

2 $$\begin{array}{@{}rcl@{}} IOU = \frac{|X\bigcap Y|}{|X\bigcup Y|}, \end{array} $$

3 $$\begin{array}{@{}rcl@{}} dice\_coe = \frac{2|X\bigcap Y|}{|X|+|Y|}, \end{array} $$

4 $$\begin{array}{@{}rcl@{}} L\_{dice} = 1 - dice\_coe, \end{array} $$

5 $$\begin{array}{@{}rcl@{}} L = L\_{dice} + \rho \left\|\vec{w}^{2}\right\|, \end{array} $$

where |*X*| denotes calculation of the number of elements in a set *X*, and $\vec {w}$ denotes the parameters of the network. We also calculate the sensitivity (true positive rate, TPR), specificity (true negative rate, TNR), and accuracy of the model on the test set, as important metrics of the performance,

6 $$\begin{array}{@{}rcl@{}} sensitivity = TPR = \frac{TP}{TP+FN}, \end{array} $$

7 $$\begin{array}{@{}rcl@{}} specificity = TNR = \frac{TN}{TN+FP}, \end{array} $$

8 $$\begin{array}{@{}rcl@{}} accuracy = \frac{TP+TN}{TP+TN+FP+FN}, \end{array} $$

where *TP* denotes the number of true positive samples (pixels), *TN* that of true negative samples, *FP* that of false positive samples, and *FN* that of false negative samples.

### Experimental results

**Fig. 4:**
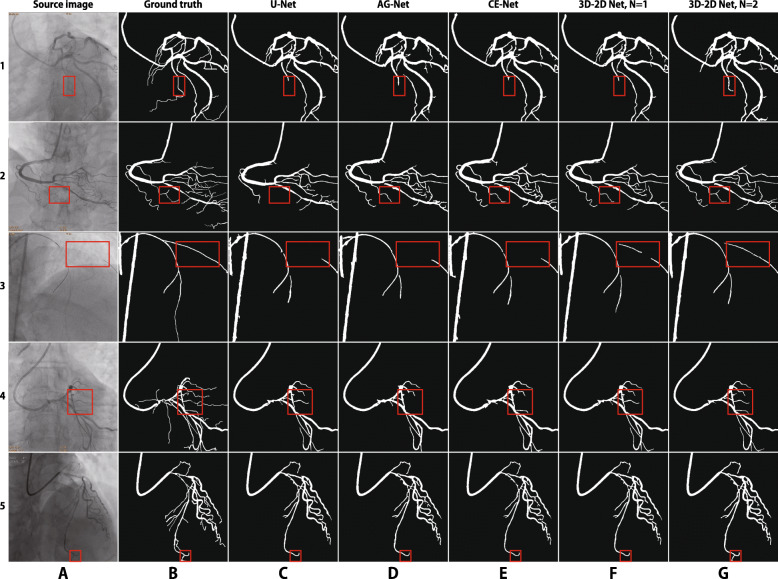
Examples of blood vessel segmentation results. Row 1 ∼5 correspond to five target images captured in five video clips, respectively. Column A: target images containing coronary arteries; col. B: ground truths, manually labeled blood vessels; col. C: segmentation results of U–Net; col.D: results of AG–Net; col. E: results of 2D CE–Net; col. F: results of our 3D–2D model, where *N* is 1; col. G: result of 3D–2D model, where *N* is 2

All the source and label images are resized to the resolution of 448×448 before being fed into the network. The four encoder modules are initiated with a Resnet–34 model pre–trained on the public ImageNet dataset. Minor modifications are made, for instance, we replace the batch normalization by instance normalization in both the encoder and decoder parts, and use stochastic gradient descend (SGD) as the optimization method. The learning rate is fixed to 2×10^−4^, and the batch size is 4. We set the parameter *N* to 1 or 2, ie., the number of input neighbor frames to 3 or 5, and train the model in 100 epochs. The output mask images are post–processed to remove the small connected components in each image and save the major parts of the mask as the segmentation result. As a comparison, we also train and test some state–of–the–art techniques, including the U–Net [9], DeepVessel [27], AG–Net [14], and 2D CE–Net [13] on our dataset. Results are illustrated in Fig. 4.

Compared with the segmentation results of the U–Net (column C), AG–Net (col. D), and CE–Net (col. E), our 3D–2D models with three and five neighboring input images (col. F and G) demonstrate better preservation capability of both the vascular details and global structures. In the third row of Fig. 4, we mark a vascular stenosis area with a red rectangle box. The contrast of this area in the source image is very low. As a result of the paucity of spatial information, U–Net, AG–Net, and 2D CE–Net fails to segment the vessel in this area. With the assistance of temporal information, our 3D–2D model using five adjacent images input successfully segments the blood vessel. Another impressive result is illustrated in the fourth row, where red rectangle boxes also annotate the difficult area. U–Net, AG–Net, and CE–Net miss a lot of fine vessels, whereas our 3D–2D network can capture some details. The more temporal information is input to the network, the higher accuracy it achieves. Similar results are observed in other images.

The performances of the models are listed in Table 1. Our 3D–2D models outperform the state-of-art techniques in almost all the five metrics. With the increasing number of the time–continuous images input into the 3D convolutional layer, the performance of the segmentation improves in a noticeable margin.

**Table 1:** Comparison of the performances of the state–of–the–art models, and the proposed 3D–2D CE–Net with *N*=1 and *N*=2 on the CadVidSet

**Model**	***N***	**sensitivity**	**specificity**	**accuracy**	**IOU (vessel)**	**IOU (background)**
U–Net (2015) [9]		0.7031	0.9798	0.9704	0.6594	0.9685
DeepVessel (2016) [27]		0.7102	0.9813	0.9722	0.6665	0.9717
AG–Net (2019) [14]		0.7256	0.9862	0.9776	0.6837	0.9765
CE–Net (2019) [13]		0.7606	**0.9943**	0.9813	0.6983	0.9845
3D–2D CE–Net (ours)	1	0.7921	0.9935	0.9854	0.7109	0.9846
3D–2D CE–Net (ours)	2	**0.7993**	0.9939	**0.9855**	**0.7137**	**0.9847**

### Implementation details

We use Pytorch to implement the proposed 3D–2D network. Using a computer equipped with an Intel i7 eighth–generation processor, NVidia RTX 2080 graphics card, and 32GB memory, the 3D–2D network proposed in this article is trained on a training set of 7363 images. The time consumed for training 100 epochs is 60 hours (*N*=1), or 68 hours (*N*=2). As a comparison, the training time of the 2D network is 50 hours. There is only 20*%*∼30*%* increase in the training time consumed. The inference time of an image is 54 milliseconds (*N*=1), 61 milliseconds (*N*=2). As the frame rate of coronary angiography video typically ranges between 10*fps* and 15*fps*, the inference speed of the proposed model fulfills the needs of real-time applications.

## Discussion

This research breaks through the barriers of traditional medical image segmentation, re–evaluates the significance of the temporal information in videos, and extends image segmentation tasks from two–dimensional configuration to spatial–temporal dimensions.

### Analysis of the results

We owe the performance enhanced by the 3D–2D network to the fact that, a 2D network is in the subspace of the 3D–2D network parametric space. Consider an extreme case: suppose that the 3D–2D network cannot learn the correlation in the time domain at all, or the image sequence has no temporal correlations between successive images at all, so the 3D convolutional kernel of the input layer has a special weight distribution – only the layer corresponding to the target image has non–zero value weights, whereas the layers corresponding to other images in the time domain have zero value weights. In this peculiar situation, a 3D convolutional kernel degenerates into a 2D kernel, and the 3D–2D network degenerates into a 2D network. This demonstrates that the 2D network is a subspace of the 3D–2D network parametric space. This implies that, theoretically speaking, the performance of the 2D network is the lower bound of the performance of the 3D–2D network for image segmentation tasks. In the absence of temporal correlations between successive images, a 3D–2D network is expected to perform as well as a 2D network, and to outperform the latter with the assistance of time–domain correlations. The experiments have confirmed that the performance of the proposed 3D–2D network is better than the 2D models. This further justifies the temporal correlations in image sequences or videos make a great contribution to the understanding of image semantic contents.

### Limitations of the method

**Fig. 5:**
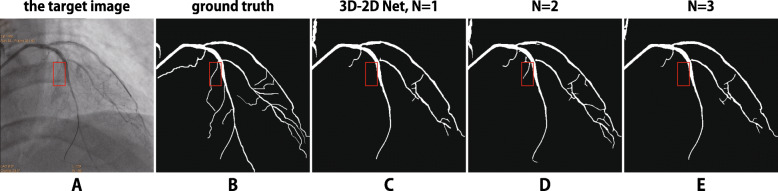
Example of a failed case with too large *N*. The best segmentation result is obtained with *N*=2 (column D), whereas the model with *N*=3 fails to extract some fine vessels (col. E)

There are still two major limitations of the 3D–2D method. The first one is the choice of *N*. *N* should not be too small that it discards sufficient time domain information, nor too large to shadow the significance of the target image and introduce time domain artifacts, as well as increase the computational complexity. As the frame rates of angiographic videos range from 10∼15*fps*,*N*≥3 is a bad choice since it means seven images are input to the model, and it covers more than half a heartbeat cycle. In the worst case this will exponentially increase the difficulty of parameter optimization, and make the network impossible to reach the optimal. One example is illustrated in Fig. 5, where *N*=2 leads to the best result, whereas *N*=3 degenerates the result.

The second limitation is that most currently available medical image segmentation datasets are not in the form of videos, but are independent images, so this method cannot be directly applied to the widely existing datasets for the time being, for example the well studied DRIVE dataset [28]. We believe that as the value of the temporal information in videos is recognized in more and more researches, video segmentation techniques will be well developed in the field of medical image analysis.

## Conclusions

By adding a 3D convolutional layer to the input layer of the 2D CE–Net to extract and fuse the temporal information in the coronary artery angiographic videos, we obtain a better performance in the segmentation tasks of the blood vessels, at a cost of a slight increment in the training and inference time. Experiments demonstrate that for the frame rate of the angiographic video, feeding five successive images into the 3D–2D network renders the best segmentation results. This work justifies that the time–domain information of videos has practical significance for image segmentation and interpretation, and is worthy of further study.

## Data Availability

Data related to the current study are available from the corresponding author on reasonable request.

## Abbreviations

2D: Two-dimensional
3D: Three-dimensional
CNN: Convolutional neural network
IOU: Intersection over union
DAC: Dense atrous convolution
RMP: Residual multi–kernel pooling
CT: Computerized tomography
fps: Frame per second
RGB: Red–green–blue
TPR: True positive rate
FPRL: False positive rate
TNRL: True negative rate
FNR: False negative rate
SGD: Stochastic gradient descend
col.: Column.
